# Drinking water quality impacts oocyte viability and embryo development

**DOI:** 10.3389/frph.2024.1394099

**Published:** 2024-08-06

**Authors:** Yasmyn E. Winstanley, Macarena B. Gonzalez, Eryk Andreas, Haley Connaughton, Jean Bergen, Miranda Ween, Darryl L. Russell, Cameron J. Shearer, Sarah A. Robertson, Rebecca L. Robker

**Affiliations:** ^1^ Robinson Research Institute, School of Biomedicine, The University of Adelaide, Adelaide, SA, Australia; ^2^ Department of Thoracic Medicine, Royal Adelaide Hospital, Adelaide, SA, Australia; ^3^ School of Medicine, The University of Adelaide, Adelaide, SA, Australia; ^4^ School of Physics, Chemistry and Earth Sciences, The University of Adelaide, Adelaide, SA, Australia

**Keywords:** water quality, female fertility, oocyte, embryo development, environmental contaminants, perfluoroalkyl and polyfluoroalkyl substances (PFAS)

## Abstract

Normal reproductive function and fertility is considered a “sixth vital sign” because disruptions to this sensitive physiological system can forewarn other health issues, including exposure to environmental toxicants. We found that female mice exhibited profound loss of embryos during pre-implantation and fetal development coincident with a change to the source of their drinking water. When female mice were provided with tap water from the building in which they were housed (Water 2), instead of tap water from a neighboring building which was their previous supply (Water 1), ovulated oocytes were degenerated or had impaired meiotic maturation, and failed to form embryos. The harmful effects of Water 2 exposure were not reversible even following a recovery period; however, carbon-filtration of Water 2 removed the toxic contaminant. Water composition analysis to identify the responsible toxicant(s) found that trace elements were present at expected levels and phthalates were undetectable. Per- and Poly-fluoroalkyl Substances (PFAS), a family of persistent organic pollutants were detected at ∼4 ng/L. To investigate further, female mice were given drinking water categorized by level of PFAS contamination (0.6 ng/L, 2.8 ng/L, or 4.4 ng/L) for 9 weeks. Compared to mice consuming purified MilliQ water, mice consuming PFAS-contaminated water had decreased oocyte quality, impaired embryogenesis and reduced cell numbers in blastocysts. PFAS concentration in the drinking water was negatively correlated with oocyte viability. Importantly, the levels of PFAS detected in the tap water are within current “safe level” guidelines, and further research is needed to determine whether PFAS are responsible for the observed reproductive toxicity. However, this research demonstrating that water deemed suitable for human consumption has detrimental effects on mammalian embryo development has important implications for public health and water quality policies.

## 1 Introduction

Safe drinking water is essential to human health and civic regulatory agencies are charged with ensuring that resident consumers are provided with water that is safe for consumption and household use. Guidelines in most municipalities dictate that water for human consumption is prevented from contacting human or livestock waste, is disinfected (e.g., chlorinated) and filtered to remove viruses where required. Higher level regulatory agencies, such as the Environmental Protection Agency (EPA), monitor potential water borne “contaminants” defined as any physical, chemical, biological or radiological substance or matter [The Safe Drinking Water Act (SDWA)]. Based on evidence of a contaminant's effects on human health and likelihood of its presence in the public water systems, new regulations or health advisories must be initiated as warranted to ensure drinking water safety (1).

Historically, there have been many instances where toxic chemicals have entered the water supply that is delivered to municipal buildings for human consumption. A particularly notorious and tragic example is the Flint Water Crisis, in which the drinking water supply to residents of Flint Michigan (USA) was switched from being provided by a treatment plant (the City of Detroit), to being piped directly from the Flint River. Soon after, Flint residents reported that exposure to the new water supply was causing skin rashes, hair loss and itchy skin, and hyperactivity and agitation in children (2, 3). It was through determined efforts of the Flint community that the toxic nature of the water supply was documented and eventually rectified (4). The importance of this episode is captured in national Drinking Water Guidelines which highlight that “Consumers are the ultimate assessors of water quality. Consumers may not be able to detect trace concentrations of individual contaminants, but their ability to recognize change should not be discounted. In some cases, consumer complaints may provide valuable information on potential problems not detected by testing water quality or monitoring treatment processes” (5).

The current study reports the findings of adverse health and reproductive outcomes in mice at a research animal facility after a change in their drinking water source. Specifically, when mice were provided with tap water from the building in which they were housed, instead of tap water from a neighboring building which was their previous supply, they exhibited skin lesions, hair loss, female infertility and fetal malformations. Notably, there was a dramatic decrease in oocyte viability and preimplantation embryo development that occurred following just a few days of exposure to the new water source. Comprehensive analysis of both water sources did not find any compounds [heavy metals, phthalates, Per- and Poly-fluoroalkyl Substance (PFAS)] that were outside of levels deemed safe. However, PFAS concentration in the mouse drinking water was significantly correlated with the loss of oocyte viability and poor embryo development. These findings, of detrimental effects on mammalian oocyte viability following exposure to tap water that is consumed by humans, have implications for public health and safe water standards in urban buildings.

## 2 Materials and methods

### 2.1 Mice

Female C57BL/6 and CBA.F1 mice at indicated ages, and males at 6–8 weeks of age, were obtained from the Western Australian Animal Resources Centre (ARC, C57BL/6 mice) or the University of Adelaide's Laboratory Animal Services (LAS, CBA.F1 mice). Mice were maintained in 12 h/12 h light/dark conditions and given water and 10% fat rodent chow *ad libitum*. The water source that mice were provided and the duration of consumption are indicated in the Results and each Figure. To determine the effect of copper levels on oocyte and embryo outcomes, mice were provided with either MilliQ water (i.e., free of trace elements) or MilliQ water supplemented with 500ppb Cu^2+^ (CuCl_2_, Chem-Supply, #CA004) for two weeks. All male mice used for sperm for IVF were provided with “Water 1”. All protocols were approved by the University of Adelaide's Animal Ethics Committee and conducted in accordance with the Australian Code of Practice for the Care and Use of Animals for Scientific Purposes.

### 2.2 *In vitro* fertilization (IVF) and embryo culture

To generate embryos via *in vitro* fertilization, female mice were given 7.5IU pregnant mare's serum gonadotropin (PMSG, Lee BioSolutions, #493-10), followed by 7.5IU human chorionic gonadotropin (hCG, Pregnyl) 47.5 h later, each via intraperitoneal (i.p.) injection. Male mice were humanely killed by cervical dislocation, and epididymal spermatozoa were collected and underwent capacitation in pre-warmed fertilization media (Vitro Fertilization; Cook Australia, Brisbane, Australia) under paraffin oil at 5% O_2_, 6% CO_2_, 37°C for 1 h prior to IVF. Female mice were humanely culled by cervical dislocation 15 h after hCG administration, and ovaries and oviducts collected and placed in pre-warmed (37°C) *α*MEM-HEPES handling media. Cumulus-oocyte complex (COC) clusters were isolated by puncturing oviducts. Following retrieval, COC clusters were gently washed twice in pre-warmed (37°C) fertilization media (Vitro Fertilization; Cook Australia, Brisbane, Australia), before being placed in a 100 µl fertilization drop containing the equivalent of 10 µl of capacitated sperm (noted as “fertilization time”), before being returned to the incubator (37°C, 5% O_2_, 6% CO_2_) for 4 h. Following this, presumptive zygotes were cleaned of all excess sperm and cumulus cells via gentle aspiration. At this time, zygotes underwent morphological and maturation assessments.

**Figure 1:**
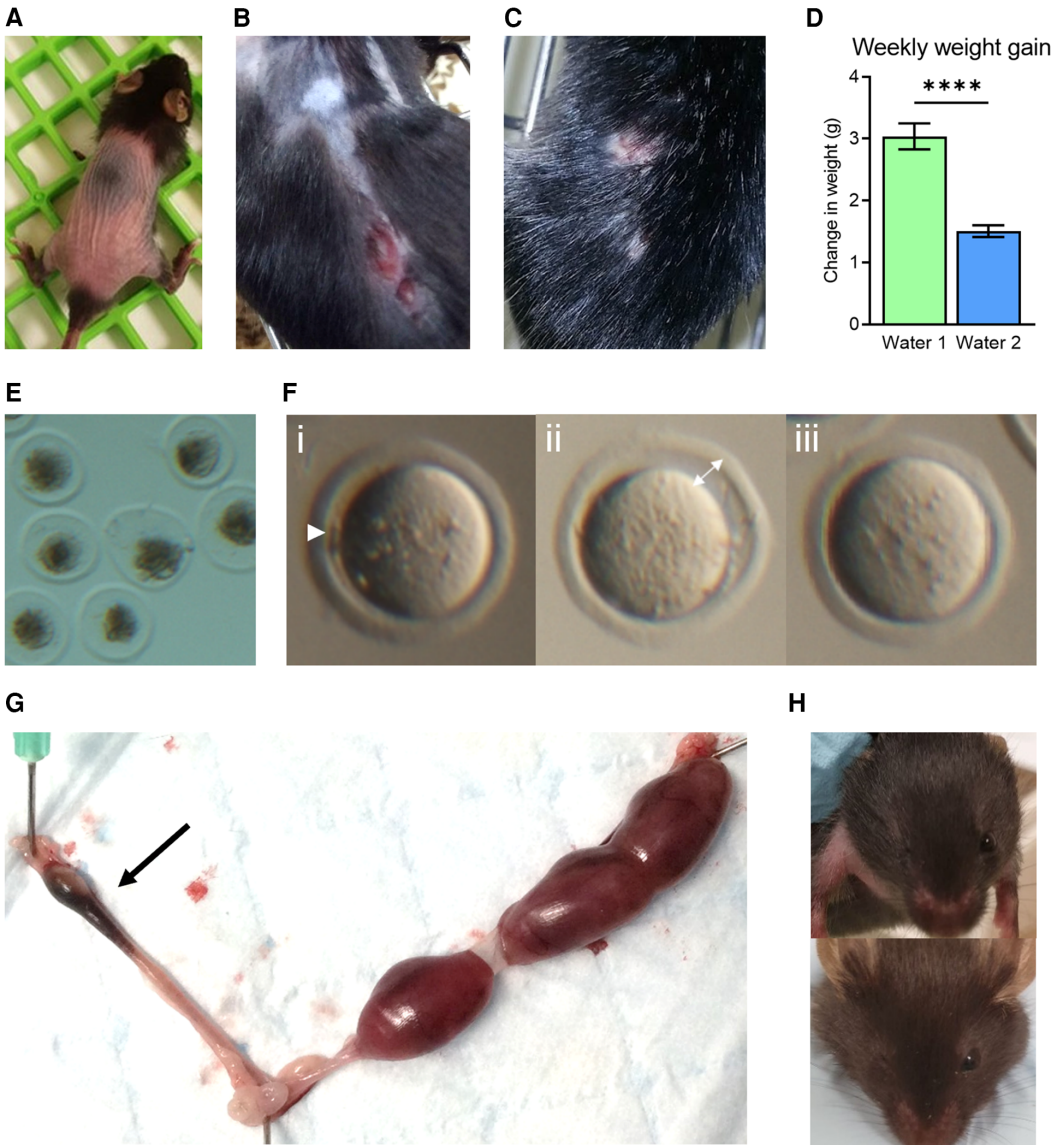
Phenotypic abnormalities observed following a facility change to water 2. Coincident with a change to the drinking water supplied to mice (Water 2), abnormal physical characteristics were noted, including hair loss in young mice (4–6 weeks, **A**), and skin lesions in older adult females (9–12 months, **B**). Aggression in males resulted in wounds from fighting (**C**). Mice used in metabolic studies exhibited a reduction in weekly weight gain (**D**). A decrease in zygote viability was observed, with many degenerated by 4 h post-fertilization (**E**). Oocytes exhibited signs of abnormal nuclear maturation, including small polar bodies (i—arrow head), increased space between the oocyte and zona (ii—arrow), and no polar body in the absence of the germinal vesicle (GV, iii) (**F**). Young female mice paired with males of proven fertility displayed elevated fetal resorption rates (arrow) as evident in excised uterus on gestation day 17.5 (**G**). Mice from breeding colonies were frequently born missing an eye, as shown in two three-week old mice where the right eye is missing (**H**).

Zygote morphology was classified into three groups: “live” zygotes displayed typical morphology, such as the oocyte occupying the majority of the zona volume, no fragmentation, and uniform cytoplasm (see Figure 3C for example), while “degenerated” zygotes appeared dark and shrunken within the zona pellucida. When extensive fragmentation was observed zygotes were classed as “fragmented” (see Figure 3D for example, degeneration and fragmentation indicated by arrows and asterisks, respectively). Zygotes were considered to have undergone normal meiotic maturation if they displayed one or two polar bodies (number of polar bodies was dependent on fertilization status, see Figure 3C, polar bodies indicated by arrow heads), and were deemed abnormal if a very small polar body or no polar body was present, despite the absence of the germinal vesicle (GV), (Figure 1G-i). This was commonly accompanied by a degree of oocyte shrinkage away from the zona (Figures 1G-ii, 3).

Live presumptive zygotes were transferred to a culture dish containing cleave media (Vitro Cleave; Cook Australia, Brisbane, Australia, 10 embryos per 20 µl cleave media drop) and returned to the incubator. For *in vivo* fertilized zygote collection, following hCG administration, females were paired 1:1 with a male of proven fertility. 16 h after hCG administration zygotes were collected from oviducts and cultured in cleave media as above. 24 h post-fertilization or post-collection, zygotes were scored as either “degenerated” (defined as morphological evidence of failed cleavage, lysis, shrinkage, and/or fragmentation), or “viable” [having successfully reached the 2 cell (2C) stage]. Embryos were then returned to the incubator until 96 h post fertilization time, when development to blastocyst stage was scored.

### 2.3 Blastocyst differential stain

Blastocysts were fixed 103 h post-fertilization in 4% PFA-PBS overnight at 4°C. Samples were washed once in PBS-PVP and incubated for 5 min at RT in 0.1M glycine, followed by 3 × 5 min washes in PBS-PVP. Samples were permeabilized in 0.5% Triton X-100 for 30 min at RT, washed 3× in PBS-PVP and blocked for 1 h at RT in 10% donkey serum (Sigma). Primary antibodies were diluted 1:500 (Oct 3/4 Santa Cruz #sc-8626, CDX2 Abcam #ab76541) in 10% donkey serum and samples incubated overnight at 4°C. Following 3 × 5 min washes in PBS-PVP, samples were incubated in secondary antibodies at a dilution of 1:1,000 for 1 h (Life technologies, Alexa Fluor 594 donkey anti-rabbit and Alexa Fluor 488 donkey anti-goat), with 10 µg/ml Hoechst-33342 added for the final 10 min of the incubation. Samples were mounted using ProlongTM Diamond Antifade Mountant (Invitrogen, #P36965) and imaged using a Cell Voyager CV1000 spinning disc confocal (Yokogawa) with a 40× objective and z-stacked with a 0.7 μm step height. Z-stacks were imported to Image J and a maximum intensity z-project was created to enable counting of individual cells.

### 2.4 Inductively coupled plasma mass spectrometry (ICP-Ms)

ICP-MS was performed on duplicate water samples from indicated sources to measure elemental content. 1.8 ml of sample was added to a 2 ml Safe Lock tube (Eppendorf, #0030123344) with 200 µl of 35% nitric acid, boiled at 96°C for 30 min, and centrifuged for 20 min at 18,000 rcf after cooling, to remove debris. Supernatants were transferred to 5 ml tubes (Sarstedt, #63.9921.522). Tubes were verified to not leach elements of interest upon acid exposure. Standards for each element were made in 3.5% nitric acid to the following concentrations (in ppb) 500, 200, 100, 50, 20, 10, 1, and blank. The full element list analyzed is: Li (lithium), Be (beryllium), Na (sodium), Mg (magnesium), K (potassium), Ca (calcium), Cr (chromium), Mn (manganese), Fe (iron), Co (cobalt), Ni (nickel), Cu (copper), Zn (zinc), As (arsenic), Se (selenium), Sr (strontium), Cd (cadmium), Sn (tin), Sb (antimony), Pb (lead), Bi (bismuth). All detected elements are shown in the figures, undetected elements are not shown.

### 2.5 PFAS content analysis

Water samples were collected in supplied plastic containers from Envirolab Services (Australia) according to Envirolab Services' instructions. “Trace level extended suite analysis” was conducted using liquid chromatography and mass spectrometry in combination (LC-MS/MS, Envirolab Services), to quantify 28 PFAS compounds with a detection limit ranging from 0.2 ng/L to 10 ng/L, depending on the PFAS compound. Detection limits for compounds found were 0.2 ng/L (PFOS, PFOA, PFHxS) and 0.4 ng/L (PFHpA, PFBS, 6:2 FTS).

### 2.6 Statistical analysis

Results are presented as mean ± SEM. Statistical analysis was performed using Graph Pad Prism version 8 for Windows (GraphPad Software Inc., La Jolla, CA). Paired *t*-test, unpaired *t*-test, one-way ANOVA, chi-squared analysis, and Pearson's Correlation were used as indicated and statistical significance was considered at *P*-value < 0.05.

## 3 Results

### 3.1 Water source is associated with physical symptoms and reproductive phenotypes in mice

Our biomedical research, focused on female fertility and embryology, utilizes mouse models which are housed within a state-of-the-art environmentally controlled animal facility, and yet suddenly, over the course of just a few weeks, mice throughout the facility exhibited highly uncharacteristic physical symptoms. These included extensive hair loss, even in weanlings, and self-inflicted flesh wounds resulting from scratching and over-grooming that were slow to heal (Figure 1A,B). Handlers also noticed unusual behaviors; particularly a high degree of agitation in young female mice, and a sudden marked increase in aggression in male mice resulting in frequent occurrences of severe fighting wounds amongst cohabitating littermates (Figure 1C). In metabolic studies where weight gain was monitored, mice exhibited an abrupt and uncharacteristic failure to gain weight (Figure 1D). Profound reproductive abnormalities were also commonly observed. Investigators studying ovarian biology found young healthy female mice ovulated degenerated oocytes (Figure 1E), and oocytes that failed to undergo normal meiotic maturation [i.e., with no polar bodies (Figure 1F)]. Mated female mice often failed to achieve pregnancy, and those that progressed to pregnancy showed poor outcomes, reflected by small litter sizes and elevated fetal resorption rates (Figure 1G). In breeding colonies, pups exhibited unusual degrees of developmental anomalies, including absence of normal eye development (Figure 1H). Whilst these observations and phenotypes were not quantified, because they occurred in multiple strains of mice and animals of both sexes, they prompted an urgent investigation into the underlying cause of these serious health issues.

**Figure 2:**
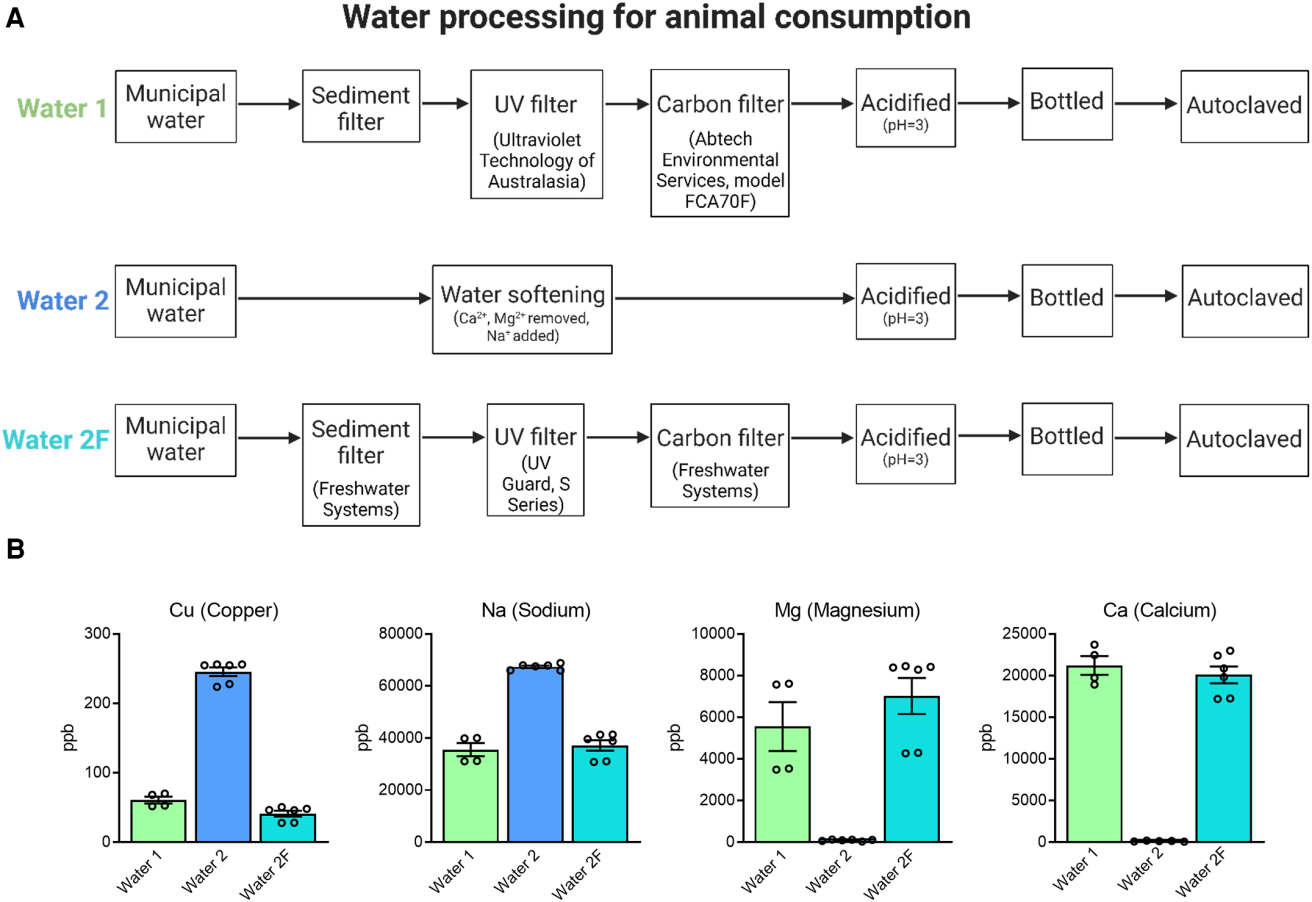
Preparation of drinking water. Animals were provided with drinking water from two different facilities (**A**). Water 1: Municipal water supplied to the building (HMAF) underwent standard procedures to sterilize water for consumption by laboratory animals, including sediment, carbon, and UV filtration, acidification and autoclaving. Water 2: Municipal water supplied to a nearby building (AHMS) underwent water softening, which adds sodium to reduce calcium and magnesium. Following observations of physical and reproductive phenotypes Water 2 processing procedures were changed to sediment, carbon, and UV filtration (Water 2F). ICP-MS analysis of mineral content in water samples from Water 1, Water 2, and Water 2F (**B**). Water 2 has notable differences in mineral content to Water 1, and Water 2F more closely reflects the composition of Water 1.

All aspects of the facility environment and husbandry were investigated as possible causes of these adverse health effects, and no alterations to diet, cages, bedding, air flow, building vibrations, or light cycles were identified. This pointed to the drinking water supply, which had been recently changed, from drinking water delivered from a neighboring facility (Water 1; which had been the source for the previous two years) to in-house drinking water (Water 2; obtained from within the facility) (see Figure 2A). Thus, we investigated whether differences between “Water 1” and “Water 2” could be responsible for the poor health phenotypes the mice displayed. Our focus was the change to oocyte quality and preimplantation embryo development, given the particular significance of environmental toxins for reproductive health. To investigate this, we utilized an experimental strategy involving generation of oocytes by gonadotropin induced ovulation, followed by *in vitro* fertilization (IVF) and *in vitro* embryo development.

**Figure 3:**
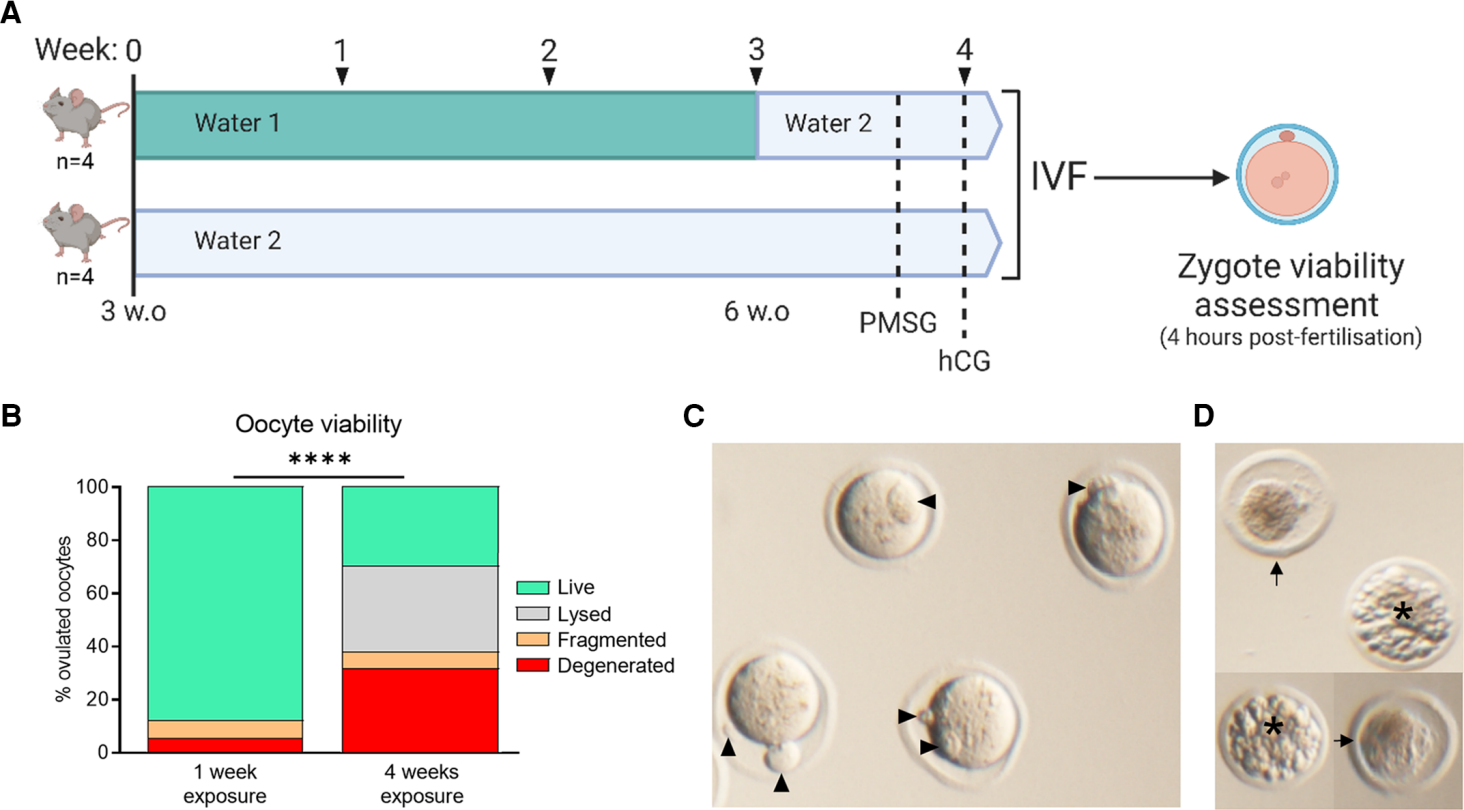
Water 2 exposure time correlates with oocyte degeneration. Mice were provided the indicated drinking water type (either 1 or 2) from 3 weeks of age. At 6 weeks of age, mice maintained on Water 1 were given Water 2 for the final week (**A**). Thus mice were exposed to Water 2 for either 1 week or 4 weeks. Ovulated oocytes were fertilized via IVF and morphology assessed 4 h later (**B**). Based on morphology, oocytes were classified as live, lysed, fragmented, or degenerated. Examples of live zygotes are shown, with polar bodies indicated by arrow heads (**C**). Zygotes classified in any category other than “live” are unable to successfully form embryos, with examples of degenerated (arrows) and fragmented (asterisk) zygotes shown (**D**). Data analyzed using chi-square test, ****p < 0.0001.

Young female mice (CBA.F1 strain, 3 weeks of age) were divided into equivalent cages and provided with Water 2 for either 1 week or 4 weeks upon arrival in the Facility, prior to gonadotropin-induced ovulation and assessment of oocyte developmental competence (see Figure 3A). Specifically, at seven weeks of age, mice were treated with gonadotropins and ovulated cumulus-oocyte complexes (COCs) were collected and fertilized *in vitro*, with zygote morphology assessed 4 h post-fertilization (Figure 3A). Mice of both groups ovulated similar numbers of oocytes but those that were given Water 2 for 4 weeks yielded greatly reduced numbers of live oocytes compared to those that had just 1 week of exposure (Figures 3B–D); due to increased proportions of degenerated oocytes (27.5% compared to 0%) and lysed oocytes (28.0% compared to 7.9%) (Figures 3B–D). As such, longer exposure to Water 2 (4 weeks vs. 1 week) resulted in a greatly decreased proportion of live, mature, and morphologically normal zygotes (see Figure 3C for examples), and a higher proportion of non-viable oocytes (lysed and degenerated, see Figure 3D for examples).

### 3.2 “Water 2” causes poor oocyte quality and impaired embryo development

**Figure 4:**
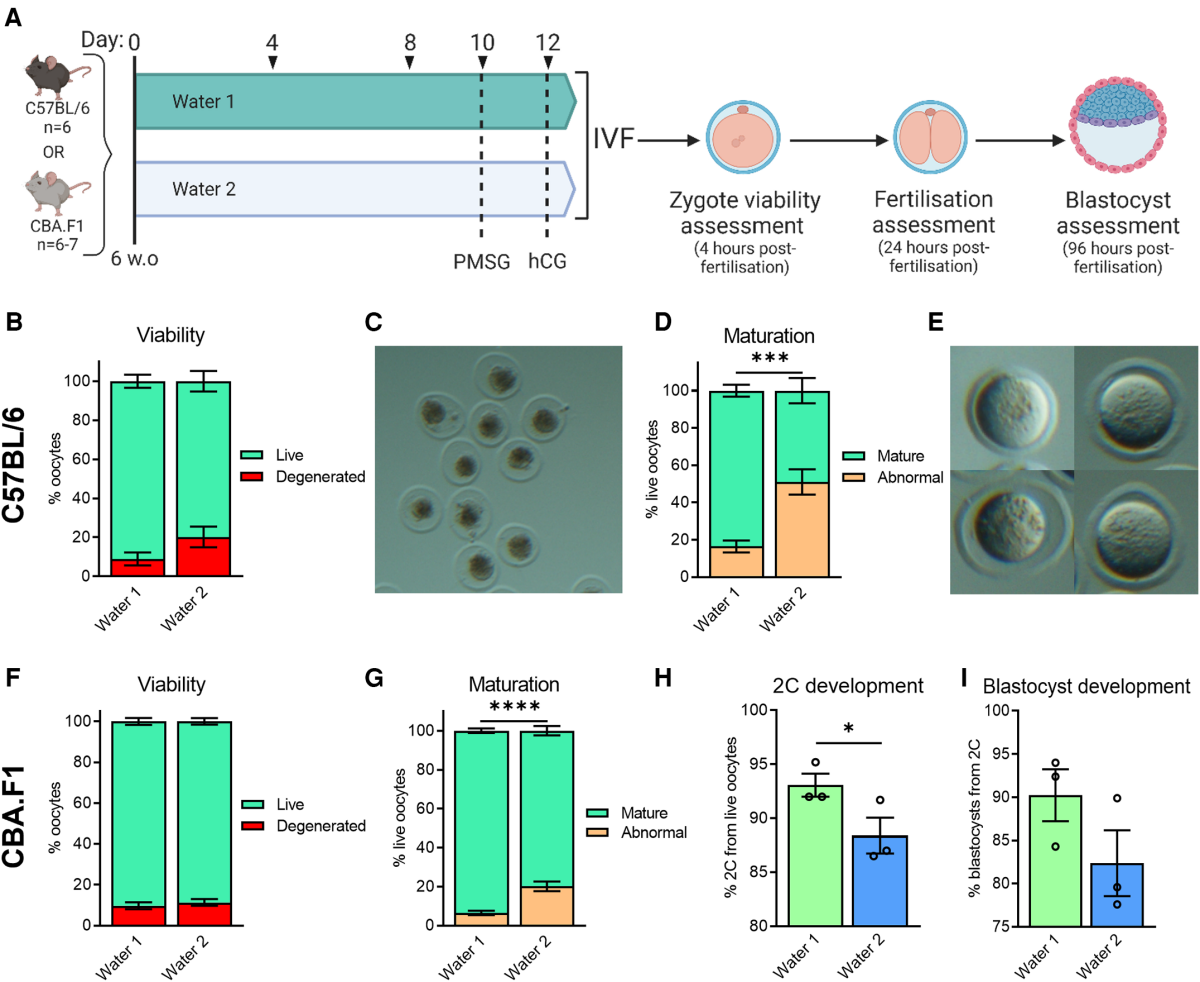
Exposure to water from different sources affects oocyte morphology, meiotic maturation, and embryo developmental competence. CBA.F1 or C57BL/6 mice were provided with either Water 1 or Water 2 for 10 days, prior to gonadotropin stimulation, resulting in a total exposure of 12 days (**A**). Ovulated oocytes were fertilized via IVF and morphology assessed 4 h later (**B–G**). Based on morphology, oocytes were classified as live or degenerated (**B,F**), with examples of degenerated oocytes shown (**C**), and live oocytes further classified as having undergone abnormal maturation or normal nuclear maturation (mature) based on polar body presence (**D,G**), with examples of oocytes without a polar body shown (**E**) In CBA.F1 mice, 2C and blastocyst development was also assessed (H and I, respectively). C57BL/6 data from *n* = 6 mice per group (**B,D**). CBA.F1 oocyte viability and maturation data from 32 mice per group (*n* = 4 independent experiments with 8 mice per group; **F,G**), and IVF was repeated *n* = 3 times with oocytes pooled from 8 mice per group each time (**H,I**). Data analyzed using unpaired *t*-test (**B–G**, where analysis was conducted on the proportion of normal oocytes, i.e., “live” for **B,F**, or “mature” for **D,G**) or paired *t*-test (**H,I**), *p = 0.0346, ***p = 0.0002, ****p < 0.0001.

To better define the period of exposure required for onset of the phenotype, and to rule out a strain-specific effect, we examined both C57BL/6 and CBA.F1 mice, at 6 weeks of age. Mice were provided with either Water 1 or Water 2 for a period of 10 days prior to commencement of gonadotropin stimulation, resulting in a total of just 12 days exposure to Water 2 (Figure 4A). In C57BL/6 mice, exposure to Water 2 did not affect the number of oocytes that were ovulated (Water 1: 26.5 ± 0.8 (*n* = 6); Water 2: 27.8 ± 0.5 (*n* = 6); *p* = 0.19 by unpaired *t*-test). However, those exposed to Water 2 displayed a trend towards poorer oocyte morphology (*p* = 0.094, unpaired *t*-test), with the mean proportion of live oocytes decreasing from 91% to 80%, and a concomitant increase in degenerated oocytes (Figure 4B). Degenerated oocytes exhibited severely abnormal morphology, and were dark and shrunken within the zona pellucida (Figure 4C). Assessment of the live zygotes showed that meiotic maturation was decreased in females exposed to Water 2 (Figure 4D) with the mean proportion of mature live oocytes only 49% compared to 84% with Water 1 exposure. Thus, mice exposed to Water 2 ovulated a high proportion of oocytes that did not possess a polar body even though germinal vesicle breakdown had occurred (see Figure 4E for examples). Further development was not assessed because the majority of oocytes were immature and not fertilizable.

In CBA.F1 mice, both groups ovulated similar numbers of oocytes (Water 1: 38.8 ± 1.2 (*n* = 6); Water 2: 35.7 ± 2.0 (*n* = 6); *p* = 0.21 by unpaired *t*-test). Zygote viability was not impacted by 12 days of Water 2 exposure (Figure 4F, *p* = 0.53, unpaired *t*-test), however the proportion of oocytes with normal meiotic maturation was decreased in mice exposed to Water 2 (Figure 4G). Subsequent development of mature fertilized oocytes to 2C embryos was diminished when mice had been exposed to Water 2 (Figure 4H), and the ability of the 2-cell embryos to form blastocysts tended to be lower compared to the Water 1 group (Figure 4I, *p* = 0.15, paired *t*-test). Taken together, these results indicate that exposure to Water 2 for just 12 days perturbs meiotic maturation in two mouse strains.

**Figure 5:**
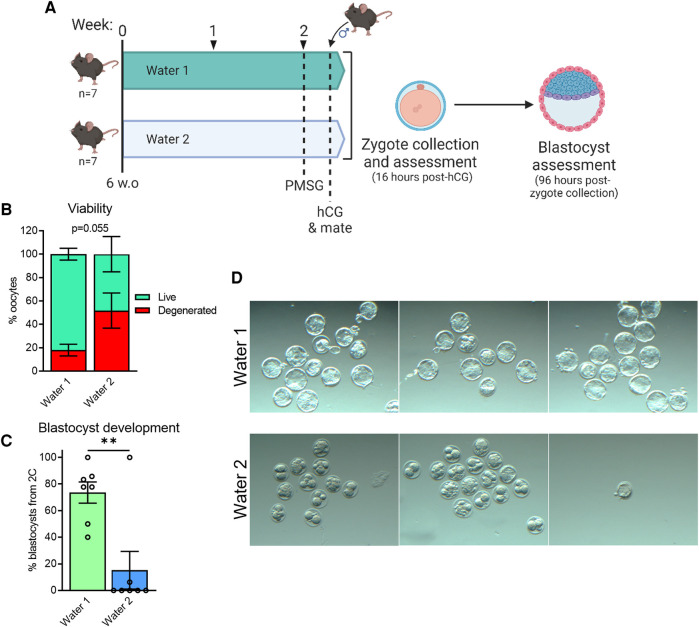
Exposure to water from different sources impacts oocyte morphology and development of embryos fertilized *in vivo*. C57BL/6 mice (*n* = 7 per group) were given either Water 1 or Water 2 for 14 days, prior to gonadotropin stimulation, resulting in a total exposure time of 16 days (**A**). After hCG administration, females were paired with male C57BL6 mice to produce *in vivo* fertilized oocytes. Based on morphology, oocytes and presumptive zygotes were classified as live or degenerated (**B**). Following 5 days of culture, the percentage of 2-cell embryos that successfully developed to blastocysts was assessed (**C**) and representative images of resulting embryos shown (**D**). Data analyzed using unpaired *t*-test **p = 0.0037.

A similar analysis was conducted on embryos fertilized *in vivo*. In this case, female C57BL/6 mice were provided with either Water 1 or Water 2 for two weeks, and then stimulated with gonadotropins to induce ovulation and housed overnight with a male of proven fertility (Figure 5A). Presumptive zygotes were retrieved from oviducts 16 h after hCG stimulation and cultured *in vitro*. Females exposed to Water 2 exhibited a high degree of oocyte degeneration (Figure 5B, *p* = 0.055, unpaired *t*-test). Following 5 days of *in vitro* culture, the proportion of 2-cell embryos that successfully reached the blastocyst stage was greatly reduced in the females exposed to Water 2 (Figure 5C), with many embryos fragmenting or arresting at early stages of pre-implantation development (Figure 5D).

**Figure 6:**
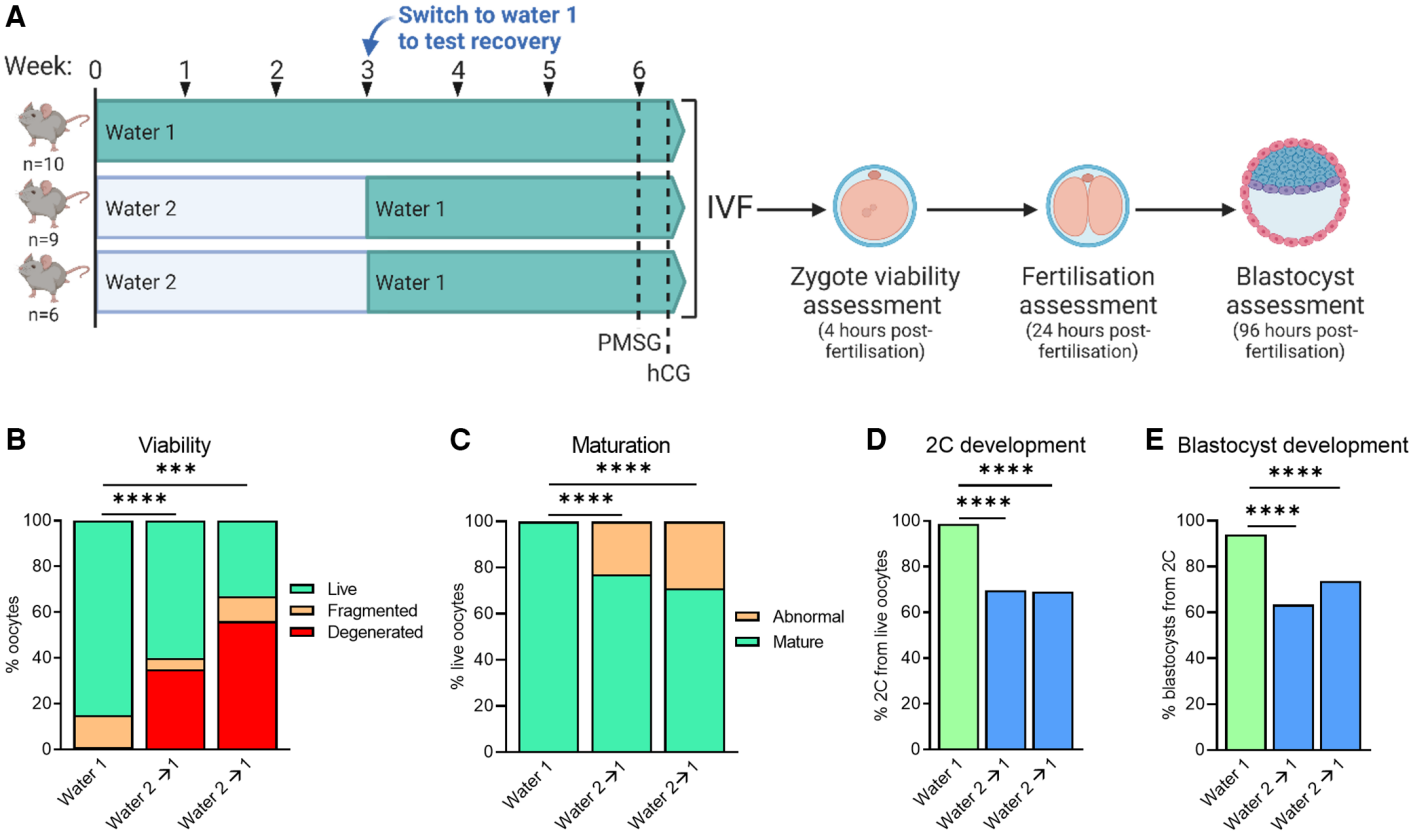
A recovery period does not rescue oocyte quality and embryo development from previous water 2 exposure. (**A**) CBA.F1 mice were exposed to Water 1 for 6 weeks. A further two groups (kept separate due to a slight age difference of 3 weeks) were exposed to Water 2 for 3 weeks, then switched to Water 1 to test a recovery period. Ovulated oocytes from each group were pooled and fertilized via IVF and morphology and maturity assessed 4 h later (**B,C**). Based on morphology, oocytes were classified as live, fragmented or degenerated (**B**), and live oocytes further classified as abnormal or mature based on polar body presence (**C**). Subsequent 2-cell and blastocyst development were also assessed (**D,E**, respectively). Data analyzed using chi-squared test, ***p = 0.0001, ****p < 0.0001.

To determine if the adverse oocyte and embryo phenotypes could be rescued in mice exposed to Water 2, the effect of a wash-out (“recovery”) period was evaluated. Some mice were provided with Water 1 for the entirety of a 6-week period, while two other groups were exposed to Water 2 for three weeks, and then switched to Water 1 for another three weeks prior to IVF (Figure 6A). Assessment of zygote morphology at the end of the fertilization period revealed that despite the recovery period, zygotes derived from mice with prior exposure to Water 2 exhibited greatly increased oocyte degeneration compared to those exposed to Water 1 (Figure 6B). The proportion of live oocytes that were meiotically mature was also markedly decreased in mice exposed to Water 2 (Figure 6C). Even in morphologically normal mature (MII stage) oocytes, 2-cell and blastocyst rates were significantly decreased in the Water 2 exposed group (Figures 6D,E, respectively). Cumulatively, this indicates that the damage induced in oocytes by exposure to Water 2 for 3 weeks is not rectified within an equivalent recovery period.

### 3.3 Carbon-filtration of water 2 improves oocyte and embryo outcomes

Considering these findings, the differences between the two drinking water sources were more closely evaluated. It was noted that Water 1 was carbon-filtered prior to its administration to mice, while Water 2 underwent a water-softening process but not carbon-filtration (see Figure 2A). Thus, the processing of Water 2 was changed to include carbon filtration, in order to mimic the purification process of Water 1, and was deemed Water 2F. This modification afforded the opportunity to investigate whether the water contained a contaminating solute that could be removed, and potentially identify this contaminant. Inductively coupled plasma mass spectrometry (ICP-MS) analysis was conducted to compare the trace-metal compositions of each water type. Analysis of mineral content showed that Water 2 had higher levels of copper, sodium, and lead, and lower levels of magnesium, calcium, lithium, strontium, iron, and chromium, compared to Water 1 (Figure 2B and Supplementary Figure S1). Modified processing to remove water softening and include UV irradiation and carbon filtration (Water 2F) restored sodium, magnesium, and calcium to levels similar to Water 1 (Figure 2B), as expected. As well, copper levels were greatly decreased in Water 2F compared to Water 2, likely due to the carbon filtration (Figure 2B). Altogether, changing the processing procedures for Water 2 to generate Water 2F resulted in a composition more similar to Water 1.

**Figure 7:**
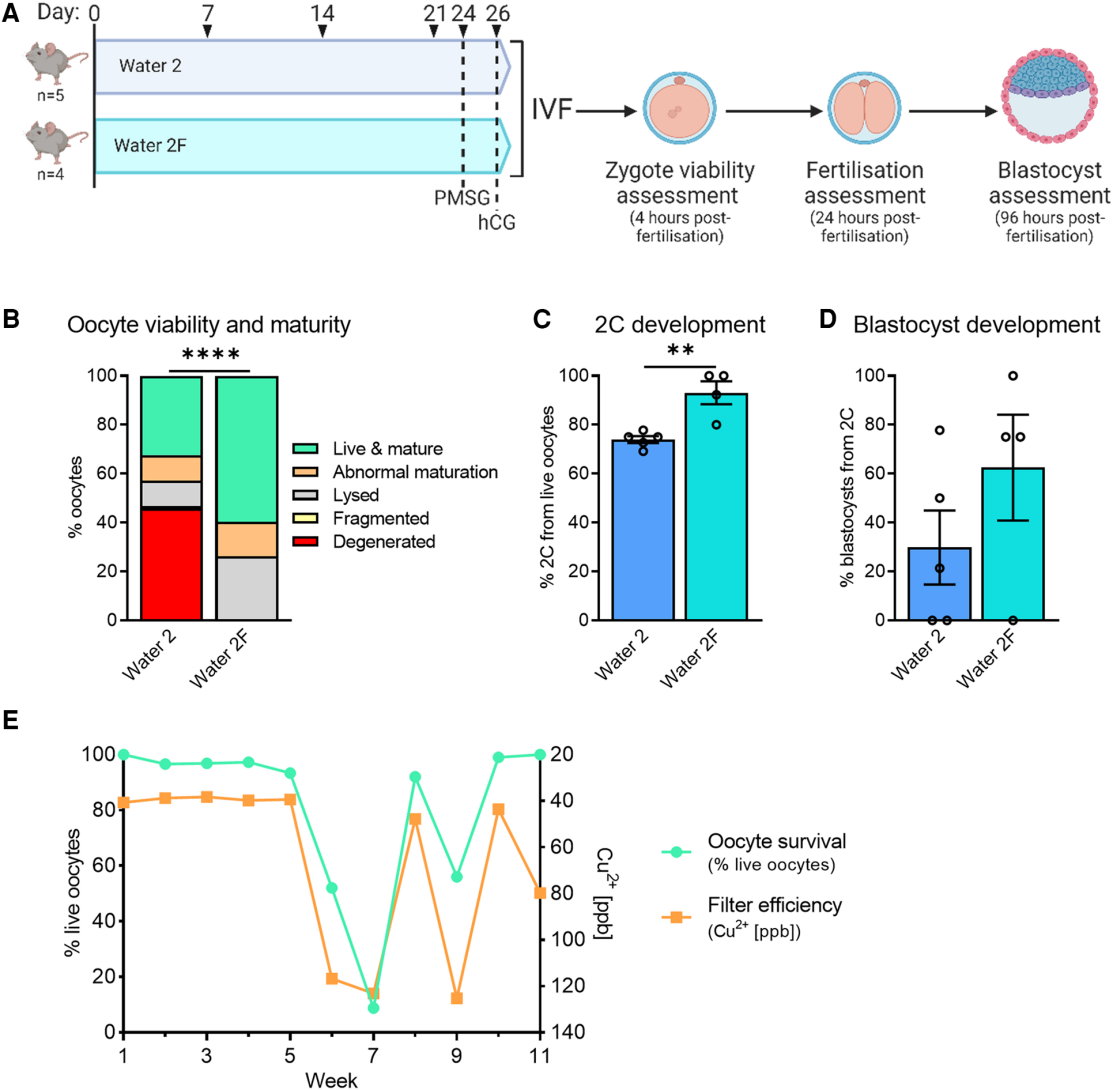
Carbon filtration of water 2 prevents toxicity to oocytes and embryos. CBA.F1 mice were given either Water 2 or Water 2F to determine if water processing via filtration could improve embryo outcomes (**A**). Four hours after fertilization presumptive zygotes were assessed for morphology and maturity (**B**). 2-cell development from live oocytes was assessed 24 h after fertilization (**C**), and blastocyst development from 2-cell embryos was assessed 96 h after fertilization (**D**). Following the transition to Water 2F, the effect on embryo development was monitored with weekly quality control IVFs. The drinking water of the mice was analyzed to demonstrate filter efficiency (orange line, right y-axis, shows copper which is removed by filtration) and compared to oocyte survival (green, left y-axis) (**E**). Data analyzed using chi-square test (**B**) or unpaired *t*-test (**C,D**), **p = 0.0036, ****p < 0.0001.

To assess the impact of the new water processing procedure on oocyte quality phenotypes, mice were provided Water 2 or Water 2F for 3 weeks prior to ovulation and IVF (see Figure 7A). Mice exposed to Water 2F ovulated a higher proportion of oocytes that were both live and mature, than females exposed to Water 2 (Figure 7B). Fertilization and subsequent 2-cell embryo development also markedly improved with filtered drinking water, by an average of 20% (Figure 7C, 73% with Water 2 vs. 93% with Water 2F), and blastocyst development occurred at normal rates (Figure 7D). Following the implementation of Water 2F for mouse consumption, quality control IVFs were performed weekly to monitor the transition and document effects on oocyte quality and embryo development (Figure 7E, Supplementary Figure S2). On the day of oocyte collections the drinking water of the mice was retained. Oocyte viability, maturity, 2-cell development and blastocyst rates were measured (Supplementary Figure S2A). Mineral levels in the mouse drinking water were analyzed as a direct functional measure of filter efficiency (Supplementary Figure S2B). Again there was a clear correlation between poor oocyte quality and mice drinking non-filtered water. Specifically, when their drinking water was appropriately filtered (depicted as Cu2+ < 100ppb), mouse oocytes exhibited typical survival rates of ≥90% (Figure 7E). In contrast, the mice that had oocytes collected in weeks 6, 7, and 9, were drinking non-filtered water, and these mice had the poorest oocyte viability. To determine if the high levels of copper in Water 2 were responsible for the reduced oocyte viability and embryo development, C57BL/6 mice were provided MilliQ water or MilliQ water supplemented with 500ppb copper for 2 weeks prior to ovulation and IVF (Supplementary Figure S3). Exposure to high levels of copper did not diminish ovulation, oocyte viability or maturity, or on-time development to 2-cell or blastocyst (Supplementary Figure S3), indicating that high copper levels were not the cause. Taken together, carbon filter-processing of Water 2 to generate Water 2F resulted in better oocyte quality and pre-implantation development. This suggested that the addition of the carbon filter removed a harmful embryo-toxic component from the drinking water.

Although they were not quantitatively assessed, other phenotypes that had been observed following the switch to Water 2 exposure also resolved following the change to Water 2F. Weight gain in mice involved in metabolic studies (Figure 1D) returned to the same rate as mice that had consumed Water 1 (Supplementary Figure S4, mean weekly weight gain: Water 1 = 3.04 g; Water 2 = 1.51 g; and Water 2F = 3.11 g). In addition, extensive hair loss was no longer observed, the high incidence of wounds disappeared, and agitation and aggression were no longer noticed. These observations in oocytes prompted analysis of sperm quality in male mice housed in the same rooms as the female mice. Sperm DNA oxidation levels, indicated by 8oxodG-positive sperm cells and indicative of DNA damage, were increased during the time when mice were maintained on Water 2, and returned to baseline levels following the change to Water 2F (Supplementary Figure S5).

### 3.4 Per- and poly-fluoroalkyl substance (PFAS) levels in drinking water correlate with poorer oocyte quality and IVF outcomes

Mineral insufficiency or imbalance was unlikely to explain the observed phenotypes as mice obtain the majority of their required mineral content from food. Also of concern was that the toxic effects on oocyte viability did not resolve when mice were switched to a water source with reduced mineral content for a further 3 weeks (see Figure 6), suggesting that developing oocytes as well as ovulated eggs were affected by the putative toxin in Water 2. Extensive testing of the water from our facilities was conducted for heavy metals (Supplementary Table S1), phthalates (Supplementary Table S2), and PFAS (Supplementary Table S3). A total of 16 heavy metals were analyzed using ICP-MS (minimum density of 5 g/cm^3^ was required to be considered a heavy metal). Many heavy metals were undetectable (cadmium, silver, thallium, tin, and uranium), or did not differ greatly between water sources (antimony, arsenic, bismuth, cobalt, manganese, and nickel) (Supplementary Table S1). Furthermore, the concentration of several detected heavy metals was not altered by the change in water processing from Water 2 to Water 2F (chromium, iron, lead, and zinc; Supplementary Figure S1) and were therefore unlikely to contribute to observed phenotypes.

Endocrine disrupting chemicals (EDCs) were also considered as a cause of water toxicity. Seven phthalate esters were analyzed and not detected in samples from either water source (Water 1 and Water 2, Supplementary Table S2). In contrast, analysis for 28 PFAS compounds detected levels of perfluorooctanoic acid (PFOA), perfluorooctane sulfonate (PFOS), perfluorohexane sulfonate (PFHxS), perfluorohexanoic acid (PFHxA), perfluoroheptanoic acid (PFHpA), and perfluorobutane sulfonic acid (PFBS) at cumulative levels of 3–5 ng/L, with PFOA, PFOS, and PFHxS being the most prevalent contaminants (Supplementary Table S3).

**Figure 8:**
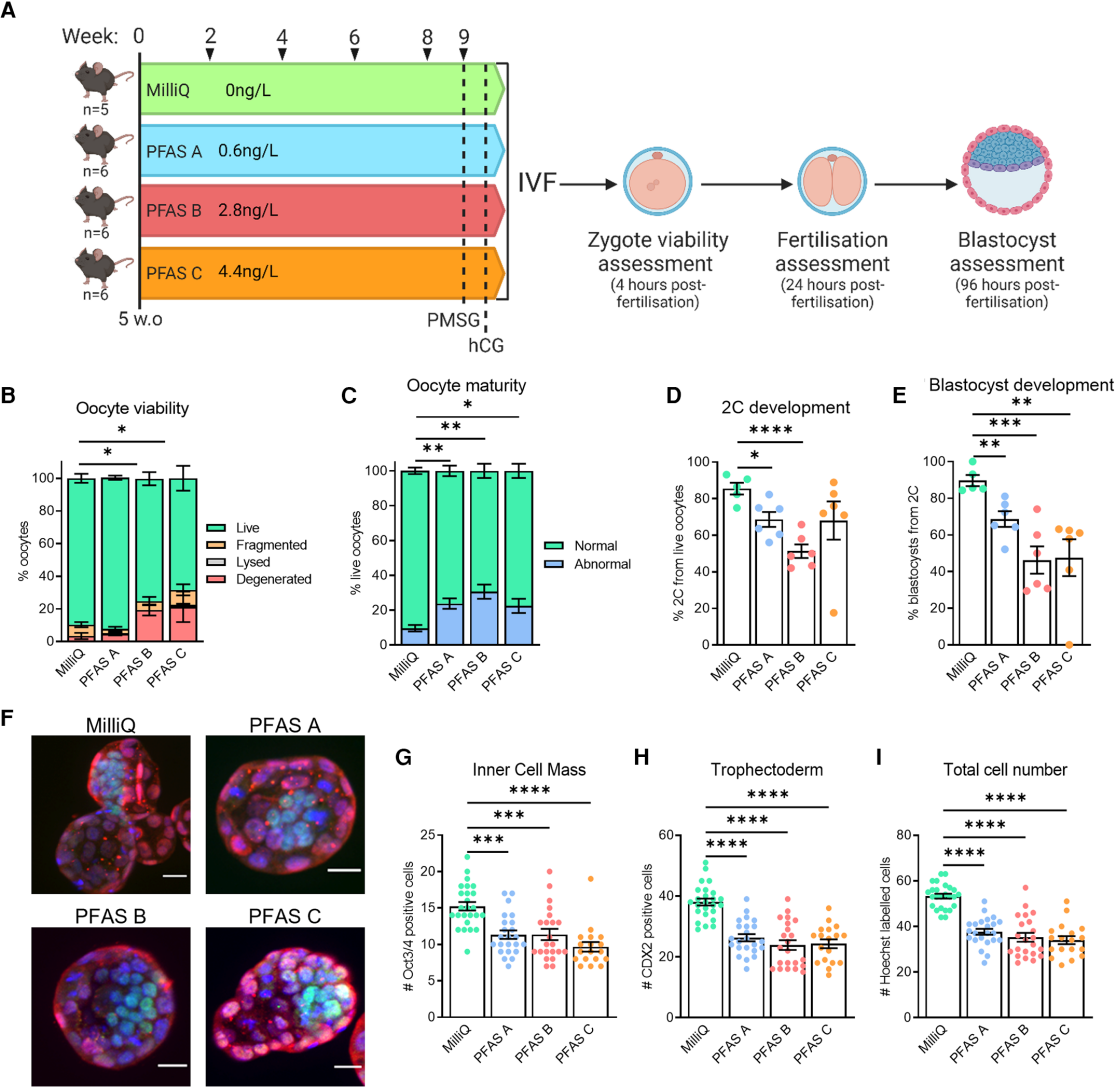
Trace-level PFAS exposure is associated with sub-fertility in female mice. C57BL/6 mice were given water from sources verified to be PFAS-free (MilliQ) or contaminated with PFAS at trace-levels (PFAS A: 0.6 ng/L, PFAS B: 2.8 ng/L, PFAS C: 4.4 ng/L) to determine if trace-level PFAS exposure was related to sub-fertility outcomes (**A**). Ovulated oocytes were fertilized via IVF and morphology and maturity assessed 4 h later (**B,C**). Based on morphology, oocytes were classified as live, fragmented or degenerated (**B**), and live oocytes further classified as abnormal or mature based on polar body presence (**C**). Subsequent 2-cell and blastocyst development were also assessed (**D,E**, respectively). Blastocysts were immuno-stained to identify the two specified cell populations and allow individual cells to be counted (**F**). Oct3/4 (green) defines the ICM (**G**), CDX2 (red) defines the TE (**H**), and Hoechst (blue) marks all cells (**I**). Data analyzed using one-way ANOVA for proportion of live oocytes (**B**), normally matured oocytes (**C**), on-time embryo development and blastocyst cell counts (**D,E,G–I**), *p < 0.03, **p < 0.005, ***p < 0.0007, **** *p* < 0.0001.

To determine if these trace levels of PFAS were contributing to sub-fertility phenotypes, mice were given either pure water (MilliQ) that was confirmed to not contain detectable PFAS, or water from the other sources (Water 1 or Water 2) for 9 weeks. For this experiment, the water sources were categorized according to their level of PFAS contamination (as PFAS A, PFAS B, PFAS C), with levels ranging from 0.6 to 4.4 ng/L (see Figure 8A). Interestingly, mice exposed to the waters PFAS B and PFAS C ovulated a slightly reduced number of oocytes (MilliQ: 29.8 ± 3.6; PFAS A: 17.7 ± 4.2 (*p* = 0.07); PFAS B: 16.5 ± 2.4 (* *p* = 0.04); PFAS C: 16.3 ± 2.4 (* *p* = 0.04); via one-way ANOVA compared to MilliQ). Ovulated oocytes were fertilized via IVF and then oocyte quality, fertilization, and blastocyst development were assessed (Figure 8A). Oocyte viability was decreased in mice exposed to PFAS B water (2.8 ng/L PFAS) or PFAS C water (4.4 ng/L of PFAS). Specifically, there was an increase in the proportion of degenerated oocytes ovulated compared to oocytes from mice exposed to PFAS-free MilliQ water or water with the lowest level of PFAS exposure (3.4% MilliQ and 4.8% PFAS A, compared to 19.4% PFAS B and 21.3% PFAS C, Figure 8B). Assessment of the live zygotes showed that meiotic maturation was decreased in oocytes from females exposed to PFAS-contaminated water (Figure 8C), with mean numbers of mature live oocytes 76.3% (PFAS A), 69.4% (PFAS B) and 77.5% (PFAS C), compared to 90.4% with MilliQ water consumption. Thus, mice exposed to PFAS-contaminated water ovulated a higher proportion of oocytes that were non-viable. Of those that were viable, PFAS exposure resulted in a higher proportion of oocytes that did not possess a polar body.

Assessment of 2-cell development rates showed a reduction in the fertilization rate for the PFAS-contaminated water groups, compared to mice that were given purified MilliQ water (Figure 8D). Further, blastocyst development assessed at day 5 post-IVF was reduced in all PFAS-exposed groups; from 89.7% in mice consuming MilliQ water, to 68.6% in PFAS A, 46.3% in PFAS B, and 47.6% in PFAS C groups (Figure 8E). To further assess blastocyst development and quality, the inner cell mass (ICM), trophectoderm (TE), and nuclei were immuno-labelled and quantified (Figure 8F). Exposure to any of the contaminated water sources (PFAS A, PFAS B or PFAS C) was associated with a decrease in the number of cells in both the inner cell mass (Figure 8G) and trophectoderm (Figure 8H) when compared to blastocysts derived from mice that had consumed MilliQ water. Overall, PFAS exposure was associated with a decreased total blastocyst cell number (Figure 8I).

**Figure 9:**
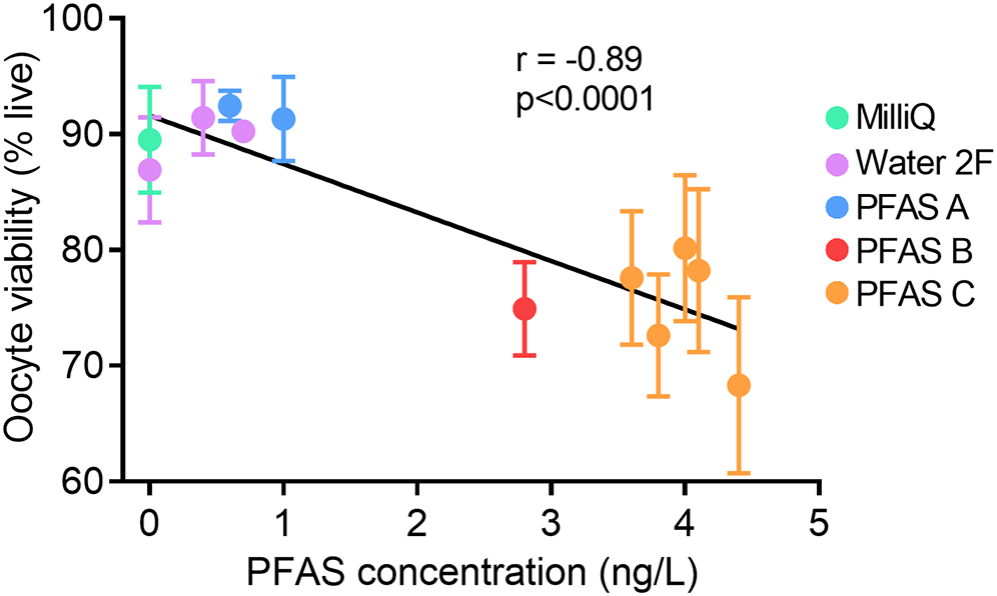
Increasing PFAS in drinking water correlates with decreased oocyte viability. Total levels of PFAS in mouse drinking water (ng/L) was correlated with oocyte viability (% live oocytes). Total PFAS concentration for PFAS A: 0.6 ng/L, 1.0 ng/L; PFAS B: 2.8 ng/L; PFAS C: 3.6 ng/L, 3.8 ng/L, 4.0 ng/L, 4.1 ng/L, 4.4 ng/L. Each data point represents the mean viability (±SEM) of oocytes from *n* = 5–6 mice. Data analyzed using Pearson's Correlation with *r*-value and *p*-value shown.

As the reduction in oocyte viability was one of the most pronounced phenotypes observed in this investigation, the proportion of live oocytes (i.e., oocyte viability rate) was correlated with the PFAS contamination level (ng/L) in water consumed by the mice, in each instance where contamination level was known (Figure 9). Increasing PFAS contamination in drinking water was associated with a clear reduction in oocyte viability. Cumulatively, these data point to PFAS as at least partly responsible for the observed adverse effects of Water 2, and implies that exposure to low levels of PFAS in water can contribute to severely compromised oocyte quality and embryo development within a matter of weeks of exposure onset.

## 4 Discussion

These observations document that two different sources of potable water, both considered safe for human consumption (5), have dramatically different effects on systemic health and female reproduction in a mammalian model system. In particular, we found that water from one building was detrimental to oocyte quality, preimplantation embryo development, and normal fetal growth in mice. Most concerning, the toxic effects on oocyte quality were not immediately reversible even when the animals were no longer exposed to the contaminated water. Future work is required to identify the specific contaminant responsible for these health effects and whether oocyte quality would eventually recover; however, reassuringly, our data shows that the toxin(s) can be removed by carbon-filtration. Amongst the panel of molecules we measured, poor oocyte quality was most significantly correlated with the levels of PFAS in the drinking water, but whether PFAS exposure is directly responsible must be addressed with future studies.

Our observation of sudden female infertility onset in response to an environmental change in an animal research facility is remarkably similar to the events that identified Bisphenol-A (BPA) as an endocrine disruptor (6). In that case, researchers noticed a sudden increase in the incidence of meiotic aneuploidy in the oocytes of their mice (7). Extensive investigations ultimately uncovered that BPA leaching from the polycarbonate plastic of the mouse cages was responsible (7). Because of this initial serendipitous observation in mice, it has now been established that BPA has serious effects on human health and female fertility (8, 9). These observations underscore the value of careful evaluation of pre-clinical models. For instance, with mice housed in research facilities, their genetic similarity across large numbers of individuals and highly controlled environmental conditions provide the opportunity to detect phenotypic changes in response to even minute exposures. The sensitivity of these and other pre-clinical models provides powerful platforms to identify factors that, similar to BPA, will emerge to also have significant effects on human health.

Our investigations have not yet conclusively identified the molecular factor(s) responsible for the multiple phenotypic abnormalities that arose in the animals exposed to Water 2, but strongly implicate PFAS compounds as at least a contributing factor. After extensive investigation of possible mediators of the detrimental health effects in mice, PFAS was identified as a compound class that was significantly different between the toxic vs. non-toxic drinking waters and correlated with poor oocyte quality. Human health effects of exposure to PFAS have been studied extensively (10–13), and carcinogenic, reproductive, endocrine, neurotoxic, dyslipidemic, and immunotoxic effects have been found. Specific epidemiological studies show PFAS exposure is associated with compromised immunity (14), impaired kidney function (15), and early menopause (16). There is a known association between serum PFAS levels and early miscarriage in women (17), and fetal loss in the affected mice was observed by multiple research teams in the building (Figure 1G and data not shown). We were also struck by the observations of eye defects (Figure 1H), that have never previously been observed in our decades of experience in breeding mice. Notably there are reports of eye malformations in children of women working in PFAS-producing factories (18), and our observations are consistent with delayed eye development in mice following high doses of gestational PFOA exposure (19).

Importantly, the PFAS levels we detected are well within standards that are deemed to be safe for human consumption. In Australia, safe drinking water guidelines for PFAS are currently set at 70 ng/L for the sum of PFOS and PFHxS and 560 ng/L PFOA by the National Health and Medical Research Council and the Natural Resource Management Ministerial Council (5). However, in June 2022 the Environmental Protection Agency (USA) announced an interim health advisory that reduced the safe drinking water level of some PFAS to 0.004 ng/L for PFOA and 0.02 ng/L for PFOS (20). The agency effectively warned that no amount of PFAS is safe. Our evidence that even short-term exposure to trace levels of PFAS in drinking water (3–5 ng/L) negatively affects embryogenesis and fetal development in mice, is consistent with this.

There are almost certainly other candidate toxic compounds besides PFAS present in Water 2 that remain to be identified. The building that housed the animals was a new construction and it is likely that plumbing materials were coated with chemicals to prevent bacterial growth, corrosion, and blockages. As evidence of this, distilled water generated on-site and plumbed to the laboratories was found to be contaminated with trace amounts of PFAS (PFOA: 0.7 ng/L ± 0.2 ng/L), and possibly other contaminants; presumably acquired during flow from the reverse osmosis equipment on the top floor of the building to the faucets several floors below. Water supplied to drinking fountains in the building had similar composition to the tap water provided to the mice: samples tested showed 414.8 ± 48.1 ppb Cu^2+^ and 3.55 ± 0.41 ng/L PFAS for instance. Whether the humans drinking this tap water experienced any cytotoxic effects is not known, but our observations raise the alarm that this is a distinct possibility.

Our work demonstrates that current regulation of drinking water standards at the level of municipal supply may be inadequate. We consider that our findings strongly point to a need for introducing strict standards and regulatory processes for water quality within the interior of buildings, particularly public buildings where large numbers of people are exposed. It is well documented that our built environments contribute to a constellation of health issues known as Sick Building Syndrome, Non-specific Building-related Symptoms (NBRS) and Building-Related Symptoms (21, 22). Yet to date the focus has been primarily on air quality, lighting, noise, and chemical contaminants such as formaldehyde and other volatile organic compounds (VOCs) (23, 24). Even in studies focused on building materials, contributions from plumbing are not included [for instance (25),]. Thus, this study contributes to the field of residential and workplace exposures and suggests that, as well as standards for water quality within buildings, better evaluation and monitoring of building materials that contact the potable water supply are required.

Lastly, this work highlights that the female reproductive system is exquisitely sensitive to environmental signals, and can be considered an “early responder” to water-borne toxicants. Future studies are needed to determine the cellular mechanisms by which PFAS, and other common environmental contaminants, are cytotoxic to oocytes and fetal tissues to a greater extent than other somatic cell types. These observations may be cause to justify advice to people who are pregnant or planning for pregnancy; specifically to include recommendations to drink carbon-filtered water where possible in order to avoid additional exposure to PFAS and other water-borne contaminants.

## Data Availability

The raw data supporting the conclusions of this article will be made available by the authors, without undue reservation.
