# Supplementary material for: Drinking water quality impacts oocyte viability and embryo development
Source: Front Reprod Health. 2024 Aug 6;6:1394099. doi: 10.3389/frph.2024.1394099 (PMC11333305; doi:10.3389/frph.2024.1394099)
Supplement: Supplementary file 1 [file Datasheet1.pdf]

Supplementary Material

1 Supplementary Figures and Tables

1.1 Supplementary Figures

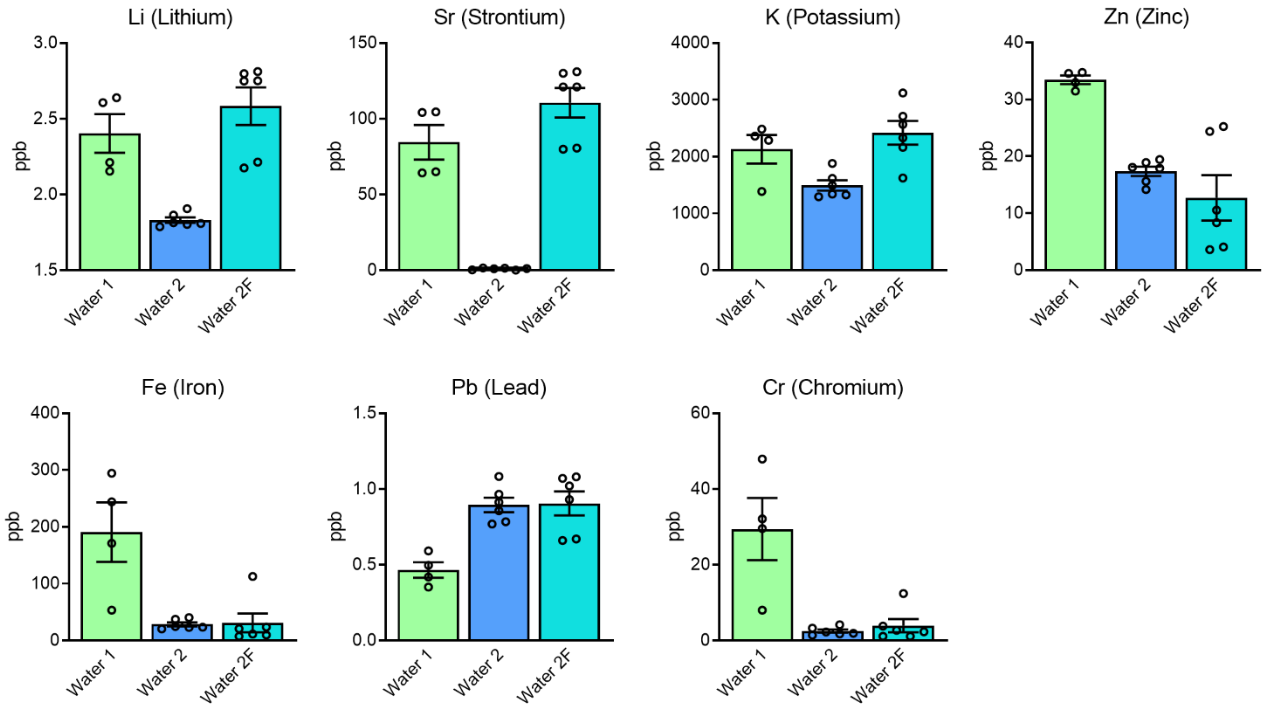

Supplementary Figure 1. ICP-MS analysis of water mineral composition. Additional mineral content from ICP-MS analysis (Figure 2) of water samples from Water 1, Water 2, and Water 2 after alterations to the water filtration and processing (Water 2F).

(A)

Weekly IVF	Filter efficiency (Cu^{2+} ppb)	Survival (%) ✓ >90	Maturity (%) ✓ >85	2C (%) ✓ >92	Blast (%) ✓ >90
1	40.62	✓	✓	✓	✓
2	38.79	✓	✓	✓	✓
3	38.26	✓	✓	✓	✓
4	39.77	✓	✓	✓	✓
5	39.36	✓	✓	✓	✓
6	116.76	✗ (52)	N/A	✗ (43)	✗ (67)
7	123.17	✗ (8.8)	✗ (38.5)	✗ (23)	N/A
8	47.75	✓	✓	✓	✓
9	125.29	✗ (56)	✗ (47)	✗ (51)	✗ (80)
10	43.54	✓	✓	N/A	N/A
11	79.78	✓	✓	✓	✓

(B)

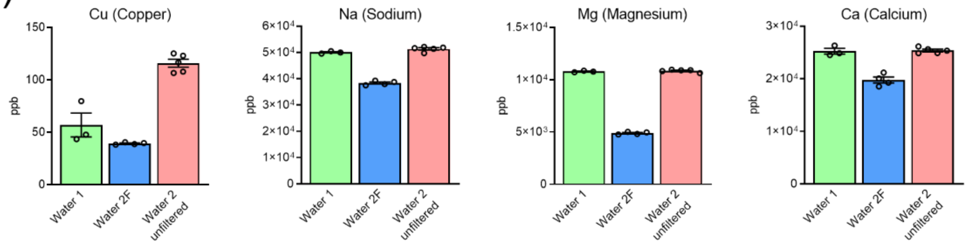

Supplementary Figure 2. Carbon filtration of Water 2 improves oocyte quality and IVF success. Oocyte quality and IVF embryo development rates were routinely assessed following modifications to water purifications systems to produce Water 2F (A). Green ticks indicate that oocyte quality and embryo development were within the indicated typical ranges. Red crosses indicate that oocyte quality and embryo development were below acceptable ranges, with the percentage of oocytes or embryos completing each milestone indicated in parentheses. N/A: not assessed. The drinking water of the mice was assessed for mineral content using ICP-MS (B) to demonstrate whether it was filtered or not. Drinking water Cu2+ levels, indicative of filter efficiency, correlate with the weekly IVF outcomes (A), specifically non-filtered water (highest Cu2+) was associated with the worst oocyte viability.

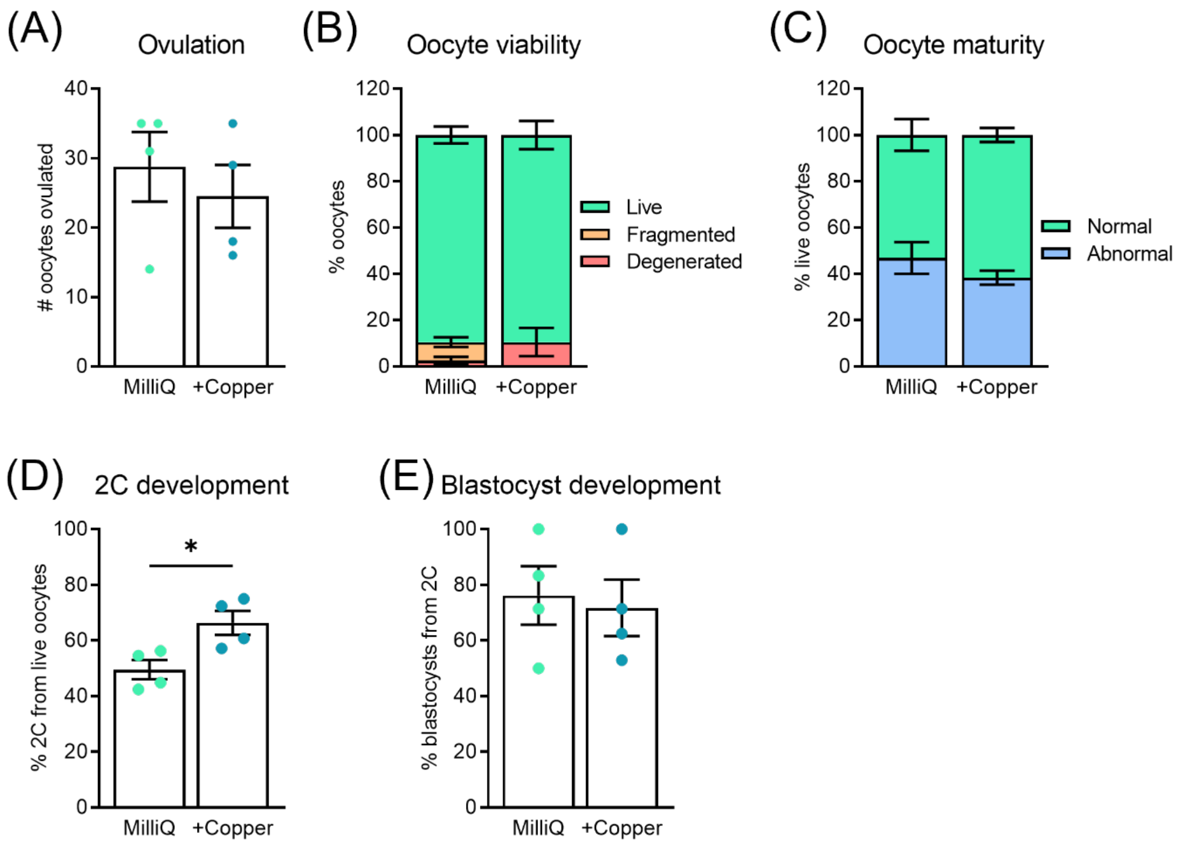

Supplementary Figure 3. High levels of copper in drinking water does not alter oocyte quality. C57BL/6 mice were given either MilliQ water, or MilliQ water with 500ppb copper (Cu^{2+}) for 2 weeks to determine if sub-fertility phenotypes were related to elevated levels of copper in drinking water. Ovulated oocytes (A) were fertilized via IVF and morphology and maturity assessed 4 hours later (B, C). Based on morphology, oocytes were classified as live, fragmented or degenerated (B), and live oocytes further classified as abnormal or mature based on polar body presence (C). Subsequent 2-cell and blastocyst development were also assessed (D and E, respectively). Data analyzed via unpaired t-test, * p=0.0234.

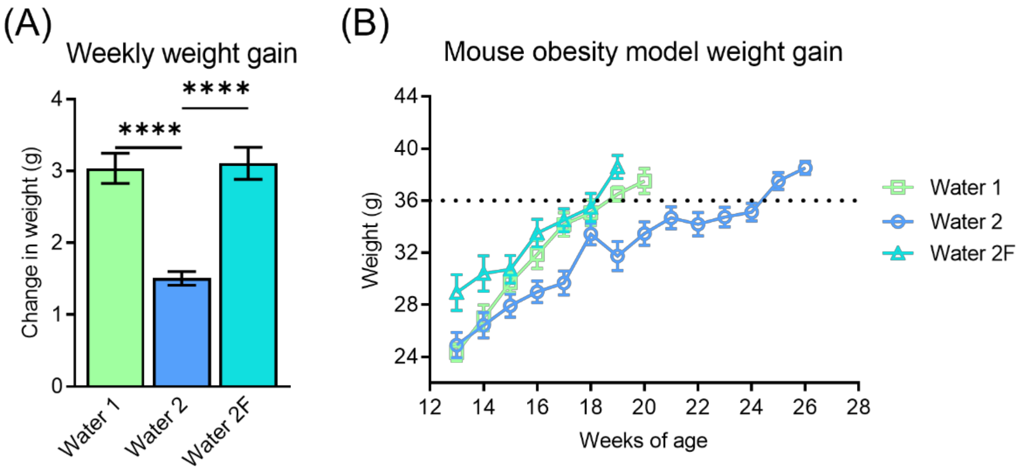

Supplementary Figure 4. Improvement of phenotypes with switch to Water 2F. Coinciding with a change to Water 2F, observed abnormal phenotypes improved. Mice used for metabolic studies that were exposed to Water 2 exhibited a reduced weekly weight gain when compared to those exposed to Water 1. When a new cohort of mice began consuming Water 2F, weekly weight gain was at comparable levels to mice that had consumed Water 1 (A). As a result of the restoration of weight gain, mice began to reach their typical weight of 36g by 19 weeks of age, as did those consuming Water 1, which was delayed until 25 weeks of age in mice exposed to Water 2 (B). Data analyzed via one-way ANOVA, **** $p<0.0001$.

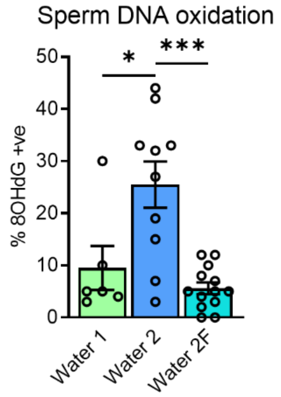

Supplementary Figure 5. Sperm DNA damage levels in mice exposed to different water sources. Routine analysis of DNA damage levels in sperm from young, healthy male mice during the time of facility water source changes showed an increase in DNA oxidation with exposure to Water 2, which decreased to baseline levels in mice exposed to Water 2F. DNA oxidation is indicated as the % of 8OHdG positive sperm per mouse. Briefly, samples were pelleted, had supernatant removed, and were resuspended in decondensation buffer (2mM DL-Dithiothreitol and 0.5% triton X-100 in PBS) for 10 minutes at room temperature (RT) before re-pelleting, supernatant removal and washing with Biggers, Whitten and Whittingham (BWW) media. Cell pellets were resuspended into BWW media and blocked using 1.5% goat serum in PBS for 1 hour at RT. Cells were re-pelleted and resuspended into 1:50 DNA/RNA damage antibody (Novus Biologicals, NB110-96878) in PBS and incubated overnight at 4°C. Following washing with PBS, cells were incubated with secondary antibody (Alexa goat anti-mouse 488, 1:400) for 1 hour at RT and washed again with PBS. Cells were quantified via manual counts under fluorescent microscopy, and those only with nuclear staining were considered 8OHdG positive. Data analyzed via one-way ANOVA, * p=0.011, *** p=0.0002.

1.2 Supplementary Tables

Supplementary Table 1. Heavy metal analysis

Element	Concentration (µg/L) Water 1	Concentration (µg/L) Water 2
Antimony	0.01 ± 0.008	0.01 ± 0.003
Arsenic	0.28 ± 0.01	0.28 ± 0.01
Bismuth	n.d.	0.07 ± 0.003
Chromium	6.91 ± 0.83	1.9 ± 0.37
Cobalt	0.1 ± 0.02	0.07 ± 0.01
Copper	33.57 ± 0.79	418.3 ± 26.15
Iron	38.95 ± 7.6	26.44 ± 3.04
Lead	0.54 ± 0.12	19.8 ± 0.16
Manganese	1.1 ± 0.27	0.65 ± 0.15
Nickel	5.23 ± 2.02	3.23 ± 0.68
Tin	n.d.	n.d.
Zinc	15.28 ± 0.72	88.32 ± 8.9

A density of more than 5 g/cm^3 was required to be considered a heavy metal. n.d. indicates 'not detected'. Cadmium, Silver, Thallium, and Uranium were also analyzed but were not detected. Detection limits for elements that were not detected were: Bismuth 0.018 µg/L, Cadmium 0.548 µg/L, Silver 0.026 µg/L, Thallium 0.012 µg/L, Tin 0.037 µg/L, Uranium 0.004 µg/L. Data presented as mean ± SEM from n=4 independent samples.

Supplementary Table 2. Phthalate Ester analysis

Phthalate Ester	Concentration (µg/L) Water 1	Water 2
Bis (2-ethylhexyl) adipate	n.d.	n.d.
Bis (2-ethylhexyl) phthalate	n.d.	n.d.
Butyl benzyl phthalate	n.d.	n.d.
Diethyl phthalate	n.d.	n.d.
Dimethyl phthalate	n.d.	n.d.
Di-n-butyl phthalate	n.d.	n.d.
Di-n-octyl phthalate	n.d.	n.d.

Detection limits for compounds were 10 µg/L (bis (2-ethylhexyl adipate) and 1µg/L (bis (2-ethylhexyl) phthalate, butyl benzyl phthalate, diethyl phthalate, dimethyl phthalate, di-n-butyl phthalate, di-n-octyl phthalate), n.d indicates 'not detected'.

Supplementary Table 3. Per- and Poly-fluoroalkyl Substance (PFAS) compound analysis in drinking water provided to mice.

		PFAS compound (ng/L)							Total PFAS (ng/L)
Source	Analysis	PFHxS	PFOS	PFOA	PFHxA	PFHpA	PFBS	6:2 FTS	
Water 2F	1	n.d	n.d	2	n.d	n.d	n.d	n.d	2
	2	n.d	n.d	0.7	n.d	n.d	n.d	n.d	0.8
	3	n.d	n.d	0.7	n.d	n.d	n.d	n.d	0.7
	4	0.2	n.d	0.2	n.d	n.d	n.d	n.d	0.4
	5	n.d	n.d	n.d	n.d	n.d	n.d	n.d	0
								**Mean**	**0.78**
MilliQ	1	n.d	n.d	n.d	n.d	n.d	n.d	n.d	n.d
	2	n.d	n.d	n.d	n.d	n.d	n.d	n.d	n.d
	3	n.d	n.d	n.d	n.d	n.d	n.d	n.d	n.d
								**Mean**	**n.d**
PFAS A	1	n.d	n.d	0.3	n.d	n.d	n.d	n.d	0.3
	2	n.d	n.d	1	n.d	n.d	n.d	n.d	1
	3	n.d	n.d	0.8	n.d	n.d	n.d	n.d	0.8
	4	n.d	n.d	0.6	n.d	n.d	n.d	n.d	0.6
								**Mean**	**0.68**
PFAS B	1	1	2	2.6	n.d	n.d	n.d	n.d	5.6
	2	1	1	1	0.4	0.9	n.d	n.d	5.1
	3	1	1	1	0.5	0.7	n.d	n.d	4.9
	4	2	1	0.6	0.6	n.d	n.d	n.d	3.7
	5	1	1	0.3	n.d	n.d	n.d	n.d	2.8
								**Mean**	**4.42**
PFAS C	1	0.7	1	1	0.6	n.d	n.d	n.d	3.3
	2	0.8	0.8	1	0.8	n.d	n.d	n.d	3.4
	3	0.9	0.7	1	1	n.d	n.d	n.d	3.8
	4	1	0.9	0.7	0.5	n.d	n.d	0.5	3.6
	5	0.9	2	0.6	0.9	n.d	n.d	0.4	4.4
	6	0.7	0.4	0.4	0.7	0.9	n.d	n.d	3
	7	1	1	1	0.8	n.d	n.d	0.5	4.6
								**Mean**	**3.73**

Detection limits for compounds found were 0.2 ng/L (PFOS, PFOA, PFHxS) and 0.4 ng/L (PFHpA, PFBS, 6:2 FTS); n.d indicates ‘not detected’.
